# DDX11-AS1 as potential therapy targets for human hepatocellular carcinoma

**DOI:** 10.18632/oncotarget.17409

**Published:** 2017-04-25

**Authors:** Min Shi, Xiao-Yu Zhang, Heguo Yu, Shi-Hao Xiang, Ling Xu, Jue Wei, Qiong Wu, Rongrong Jia, Yu-Gang Wang, Xiao-Jie Lu

**Affiliations:** ^1^ Department of Gastroenterology, Shanghai Tongren Hospital, Shanghai Jiao Tong University School of Medicine, Shanghai, China; ^2^ Department of General Surgery, Division of Gastrointestinal Surgery, The Affiliated Huai’an Hospital of Xuzhou Medical College and The Second People's Hospital of Huai’an, Huai’an, Jiangsu, China; ^3^ NPFPC Key Laboratory of Contraceptives and Devices, Shanghai Institute of Planned Parenthood Research (SIPPR), Institutes of Reproduction and Development, Fudan University, Shanghai, China; ^4^ Department of General Surgery, Liver Transplantation Center, The First Affiliated Hospital of Nanjing Medical University, Nanjing, Jiangsu, China

**Keywords:** liver hepatocellular carcinoma, lncRNAs, DDX11-AS1

## Abstract

Hepatocellular Carcinoma (HCC) is one of the most fatal cancers, whose incidence and death rates are still rising. Here, we report the identification of long non-coding RNAs (IncRNAs) that associated with HCC progression and metabolism based on the systematically analysis of large scale RNA-seq data from HCC patients. We identified seven lncRNAs with high confidence which were highly related with prognostic of HCC. Of note, three of them had quite different expression patterns between the control samples and the patients, and their critical roles in cancer progression were validated. We proposed that *DDX11-AS1* play important role during HCC oncogenesis and may serve as potential therapy target for HCC.

## INTRODUCTION

It was estimated that there would be over 39,000 new hepatocellular cancer (HCC) cases and over 27,000 deaths caused by it in the United States in 2016 [1]. Contrary to decreasing trends for most cancers, join point analysis suggested that incidence and death rates rose in both sexes for liver cancers, which is one of the most fatal cancers, from 2003 to 2012 [1]. It prevalence and mortality urged us to find out the factors affecting its incidence and prognosis.

Long non-coding RNA (lncRNA) is a massive and diverse class of non-coding RNA genes. The lncRNAs may perform as a platform for the complicated interaction with miRNA, mRNA, protein or their complex, and they also worked as an essential regulator in almost every aspect of human body. Studies have indicated that dysregulated expression of lncRNAs had impacts on the capacities of cancer cells for tumor initiation, growth, and metastasis, which served them as promising targets for cancer diagnosis and therapy [2]. However, there were only several lncRNAs that have been functionally annotated, the majority still remains to be characterized. Different high-throughput methods to identify new lncRNAs (including RNA sequencing and annotation of chromatin-state maps) have been applied in various studies searching the potential therapy target for cancers. However, the potential role of lncRNAs that play in the progress of HCC remains unknown. Identification of the critical lncRNAs which associated the LHC would provide new insight for discovery of new therapy.

In this study, we studied the influence of lncRNAs on the liver cancer initiation and progression taking advantage of the large scale of RNA-seq data. We first constructed one high confidence lncRNAs expression dataset by link TCGA liver hepatocellular carcinoma dataset and a curated lncRNA databasets. Furthermore, the differentially expressed lncRNAs in the patients and their biological functions were studied.

## RESULTS

### The differentially expressed lncRNAs

**Figure 1:**
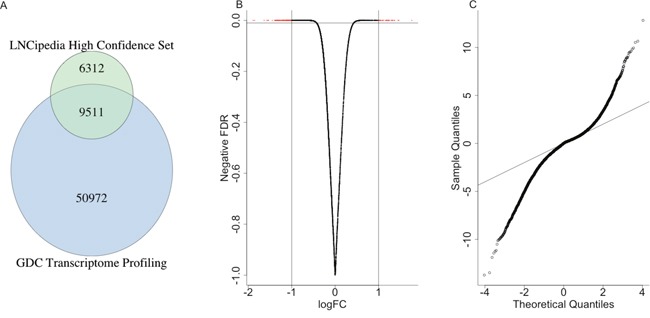
The differentially expressed lncRNAs **(A)** The venn diagram indicated the intersection of expression level data from GDC and high confidence lncRNAs. **(B)** The volcano plot showed the correlation between logFC and the negative odds. **(C)** The QQ-plot indicated the distribution of FDR of differentially expressed lncRNAs, which suggested many more lncRNAs were significantly differentially expressed compared with random situation (the black line).

We obtained expression levels of 60483 genes from TCGA liver hepatocellular carcinoma patients and a high confidence lncRNA sets from LNCipedia [3, 4]. The intersection of them gave us totally 9511 high confidence lncRNAs to finish the following analysis (Figure 1A).

Totally, 1736 lncRNAs were differentially expressed between the control sample and the patients with a p value less than 0.01 (Figure 1B), where 452 lncRNAs were highly expressed in the control samples and 1284 were highly expressed in patients. The Q-Q plot was shown in Figure 1C, which indicated that many more lncRNAs were significantly differentially expressed compared with random situation. It suggested that the expression levels of many lncRNAs were highly related with cancer occurrence and progression, either drove the cancer or were influenced by other micro-environment molecules. Such aberration from the expected line also suggested that the important roles of lncRNAs played in the liver cancer patients.

### The expression patterns of lncRNAs

**Figure 2:**
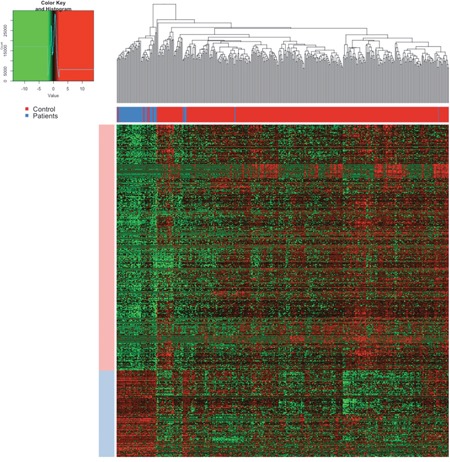
The heatmap of lncRNAs The samples were represented in columns and the lncRNAs were represented in columns. In the upper, the blue color annotated the control samples and the red one annotated the patients, which was separated quite well according to the dendrogram. In the center of the heatmap, the red meant the high expression and green meant the low expression as annotated in the color key.

The expression levels of these differentially expression lncRNAs were plot as heatmap (Figure 2). The samples were represented in columns and the lncRNAs were represented in columns. The control samples were shown as blue and the patients were shown as green color on the column side, which was separated quite well according to the dendrogram. If the dendrogram was cut into two groups, the accuracy of separating the control samples and the patients was over 90%.

In the center of the heatmap, the red meant the high expression and green meant the low expression as annotated in the color key. Notably, the expression levels of lncRNAs were quite stable in the control samples after normalization but were relatively heterogenic. The group of highly expressed in the control samples were annotated as light blue and that of lowly expressed as light red. Such heterogeneity of the expression levels of lncRNAs in the patients suggested the instable regulation and their important roles in cancer progression.

**Figure 3:**
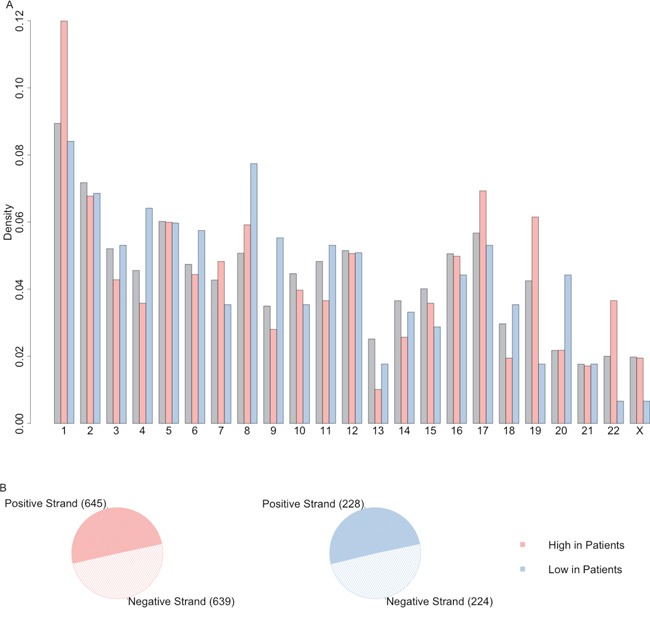
The distribution of differentially expressed lncRNAs on chromosome and strand **(A)** The distribution of lncRNA of up-regulated lncRNAs (light red) and down-regulated lncRNAs (light blue) across the chromosome, compared with distribution of all the high confidence lncRNAs (grey). **(B)** The distribution of lncRNA of up-regulated lncRNAs (light red) and down-regulated lncRNAs (light blue) on positive or negative strand.

The distribution of lncRNA of up-regulated lncRNAs and down-regulated lncRNAs across the chromosome and between positive or negative strand were plotted as Figure 3. These differentially expressed lncRNA were located evenly on positive and negative strands but they had different patterns on the chromosomes. For example, relatively higher percentage of up-regulated lncRNAs were located on the chromosome 1, 17 and 22. On the other hand, down-regulated lncRNAs had higher rate to be on chromosome 8 and 20 compared with all the high confidence lncRNA datasets. It suggested that some lncRNAs may have structural correlation in some chromosome.

### The performance of top differentially expressed lncRNAs

**Figure 4:**
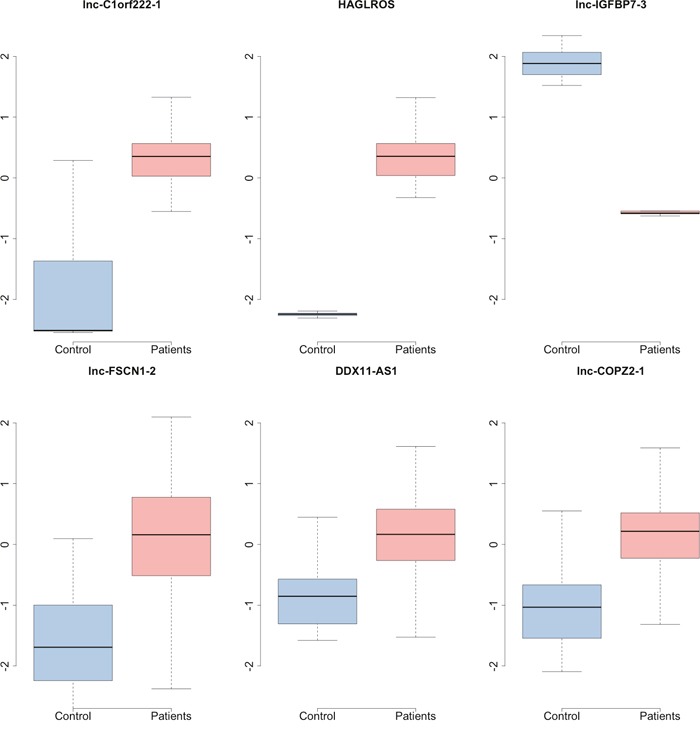
The boxplot of top lncRNAs which were differentially expressed between the control samples and parients with hepatocellular carcinoma

After p value adjustment with FDR, 7 top lncRNAs with a FDR less than 0.5 were filtered out into next analysis (Table 1). It seemed that these three genes had quite different expression patterns between the control samples and the patients (Figure 4).

**Table 1:** The performance of top differentially expressed lncRNAs

Symbol	Ensemble Gene ID	logFC	P Value	FDR
lnc-C1orf222-1	ENSG00000233542.1	0.2647883	2.83E-07	0.002080578
HAGLROS	ENSG00000226363.3	0.2600972	4.57E-07	0.002080578
lnc-IGFBP7-3	ENSG00000251049.2	−0.2473662	1.61E-06	0.004896557
lnc-FSCN1-2	ENSG00000230733.2	0.2293141	8.72E-06	0.019858126
DDX11-AS1	ENSG00000245614.3	0.2205766	1.89E-05	0.034469534
lnc-COPZ2-1	ENSG00000263412.1	0.2171767	2.54E-05	0.038541446
lnc-IL17RC-2	ENSG00000269894.1	0.2152517	2.99E-05	0.038946373

We also tried to fit the COXPH model to the study the relationship between the expression levels of lncRNAs and the survival status of patients. Among the 7 top lncRNAs, only *DDX11-AS1* had a significant association with survival time with a p-value of 0.0117.

However, the limited studies about these lncRNAs restricted the functional analysis. An alternative method to learn about their functions was to study the functions of their co-expressed mRNAs. Due to the large volume of potential co-expressed mRNAs, we limited our slope to the top 3 lncRNAs. The top KEGG pathways were listed in Table 2 and the full tables of KEGG pathway and GO term enrichment are available in the Supplementary Table 1 and Supplementary Table 2 due to their sizes. With regard to the enriched pathway or GO terms, these lncRNAs were highly correlated with cancer, metabolism and DNA transcription and translation. These results supported our hypothesis that these lncRNAs played important roles in the liver hepatocellular carcinoma initiation and progression.

**Table 2:** The Kegg-pathway enrichment of co-expressed mRNAs

Accession	Pathway Name	P Value	Bonferroni
hsa05200	Pathways In Cancer	0	0
hsa03040	Spliceosome	0	0
hsa05016	Huntingtons Disease	0	0
hsa04120	Ubiquitin Mediated Proteolysis	0	0
hsa03010	Ribosome	0	0
hsa00970	Aminoacyl Trna Biosynthesis	1.19E-12	2.15E-10
hsa04510	Focal Adhesion	4.51E-11	8.12E-09
hsa00240	Pyrimidine Metabolism	6.94E-11	1.24E-08
hsa04144	Endocytosis	1.02E-10	1.83E-08
hsa04110	Cell Cycle	1.08E-10	1.95E-08

The curated lncRNAs were retrieved from database Lnc2Cancer [5] and paper of Su, Malouf [6]. Notably, most of the curated lncRNAs were not presented in our 9511 high confidence lncRNA dataset. Only liver cancer related lncRNAs from Lnc2Cancer were extracted. The final list of cancer-related lncRNAs which were also presented in our lncRNA pool was list as Supplementary Table 3. None of these six curated lncRNAs was significantly differentially expressed in the liver cancer. Strangely, HULC, which was named as highly up-regulated in liver cancer, was not significantly differentially expressed (Supplementary Table 3).

## DISCUSSION

The curated lncRNAs were retrieved from database Lnc2Cancer [5] and paper of Su, Malouf [6]. Notably, most of the curated lncRNAs were not presented in our 9511 high confidence lncRNA dataset. Some of them did not belong to the high confidence lncRNAs, the identification of some lncRNAs could be converted into ensemble gene id and others’ expression levels were unavailable. This hinted the complicated and disordered database and studies for lncRNAs.

In spite of the important effects of lncRNAs *in vivo*, the majority of lncRNAs did not have any functional annotation. Luckily, with the help of RNA sequencing and chromatin-state map, multiple unrelated lncRNA datasets were built independently, like LNCiepdia [3], NONCODE [7], lncRNAdb [8] and so forth. They also had their own naming system which is hard to be converted. Some naming/id system of lncRNAs included HUGO approved names, which only contained 214 genes, ensemble gene id, LNCipedia naming system, NONCODE [7] naming system, and even microarray probe IDs. These disordered lncRNAs’ databases give a foundation for lncRNA studies but also make their studies more complex.

Besides the complex databases, there were few phenotypic information about the lncRNAs. Though a lot of common SNPs were found for the differentially expressed lncRNAs, there were no OMIM allelic variant SNPs were available till now. For example, the lncRNA HAGLROS had a lot of items in ClinVar and dbVar which suggested that it might have pathogenic effects but no real studies had studied it by experiments. More attention is needed on the study of lncRNAs.

Due to the limited systematic studies about lncRNAs, we failed in finding the known function of the cancer-related lncRNAs on our top list. On the other hand, we tried to obtain the co-expressed mRNAs to figure out the possible function of these lncRNAs. Considering the large size of the available mRNA numbers even after p value adjustment, we only used the top three differentially expressed lncRNAs to learn their biological functions. The results showed that these lncRNAs were correlated with pathways in cancer, DNA repair and metabolisms (Table 3 and Supplementary Table 1, Table 2). This is a good evident to support our results that lncRNAs had important effects on the cancer progression.

**Table 3:** The structural variants on lncRNA DDX11-AS

ID	Variation Class	Type	Consequence	Location
esv3629033	CNV	Loss	intron variant; non coding transcript variant	12:31038542–31040215
esv3629034	mobile elementinsertion	intron variant; non coding transcript variant; feature elongation	12:31061589	
esv3629035	CNV	Loss	intron variant; non coding transcript exon variant; non coding transcript variant	12:31071531–31073210

Though only a few studies of the differentially expressed lncRNAs on cancer were found, some side evidences were able to support their function in the liver cancer. For example, lnc-IL17RC-2 participated in T helper 17 mediated inflammatory responses, which with a large possibility was stimulated by the cancer initiation [9]. Kelemen, Lawrenson [10] reported several risk associations for ovarian carcinomas in genome-wide association studies and HAGLROS was located in one of the regions. Another two studies identified susceptibility loci for ovarian cancer where was HAGLROS located [11, 12]. However, there was no further evidence to indicate whether HAGLROS affected the cancer directly or was only passenger of other cancer-related genes. Marchese, Grossi [13] renamed DDX11-AS1 as cohesion regulator noncoding RNA (CONCR). It could be activated by MYC and up-regulated in multiple cancer types, and play direct role in the establishment of sister chromatid cohesion by modulating DDX11 enzymatic activity.

**Figure 5:**
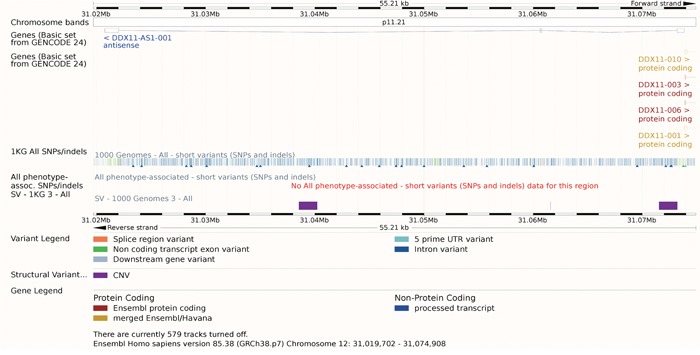
The gene model of DDX11-AS1 Though there were many SNPs or indels annotated, none of them was linked with phenotypes. The track SV – 1KG 3 - ALL showed that three CNV appears in the region of DDX11-AS.

The survival analysis showed that the expression level of DDX11-AS1 were associated with the survival status. As a result, we would like to look more carefully at DDX11-AS1 (Figure 5). The official full name of DDS11-AS1 is official full name DDX11 antisense RNA 1, which had three transcripts. Marchese, Grossi [13] renamed DDX11-AS1 as cohesion regulator noncoding RNA (CONCR). It could be activated by MYC and up-regulated in multiple cancer types. They found that the decreasing expression level of DDX11-AS1 led to lower cellular proliferation, increased apoptosis and hindered the cell at G0/G1 stage. DDX11-AS1 is the antisense RNA of DDX11, but it did not regulate the mRNA expression level or protein levels of DDX11. On the other hand, it modulated the activity of the helicase DDX11. They proved that DDX11-AS1 is critical for DNA duplication and play direct role in the formation of sister chromatid cohesion by regulating the enzymatic activity of DDX11.

Except for this detailed study, no other study talked about the function of DDX11-AS1. As a result, though there were many SNPs or indels annotated, none of them was linked with phenotypes. There were three Copy Number Variants (CNV) appears in the region of DDX11-AS as the purple rectangle showed. These CNV had effects on this lncRNA in many aspects (Table 3). Considering its critical role in the DNA duplication, these CNV might aim the cancer progression, which needs further studies.

Here, we proposed that the seven lncRNAs in our top list, including *lnc-C1orf222-1, HAGLROS, lnc-IGFBP7-3, lnc-FSCN1-2, DDX11-AS1, lnc-COPZ2-1 and lnc-IL17RC-2*, played important roles in the liver hepatocellular carcinoma, whose dysregulated expression levels led to cancer progression. Especially, *DDX11-AS1 was* suggested as potential therapy targets for LHC and worth studying thoroughly.

## MATERIALS AND METHODS

### TCGA hepatocellular carcinoma dataset

The Cancer Genome Atlas (TCGA) datasets, including clinical information and RNA-seq results of patients with liver hepatocellular carcinoma based on Illumina Genome Analyzer RNA Sequencing, were retrieved from Genomic Data Commons (GDC) Data Portal on July 24^th^, 2016. For mRNA-Seq data, the GDC generated gene level and exon level quantification in Fragments Per Kilobase of transcript per Million mapped reads (FPKM) and provides Upper Quartile normalized FPKM (UQ-FPKM) values using GRCh38/hg38 as the reference genome. In this study, UQ-FPKM values were used as the inputs. This dataset contained 377 patient cases and 52 control samples. There were expression levels of total 60483 different coding or non-coding genes.

### High confidence lncRNA datasets

LNCipedia is an integrated database of 118,777 human annotated lncRNA transcripts obtained from different sources [3, 4]. There were totally 15823 lncRNAs annotated as high confidence in LNCipedia which were used as the lncRNA pool in this study. The ensemble gene IDs were extracted from GTF file annotated based on the reference genome GRCh38/hg38. The intersection of the ensemble genes from the GDC Data Portal and the highly confident lncRNAs were extracted and 9511 genes were obtained for the next steps (Figure 1).

### Differentially expressed lncRNAs

The expression levels of extracted 9511 lncRNAs were scale to the z-score across all the genes according to the following equation first:

$$z=\frac{x-\mu }{\sigma },$$

where μ is the mean of expression levels of each lncRNA and σ is the standard deviation. Using the scaled expression levels, R package limma [14] was used to identity the differentially expressed genes between the control group and the patients. Moderated t-statistics is used to for significance analysis where the standard errors were moderated across genes using the Bayesian model [15]. This step borrowed information from the collection of genes to help with inference about every individual gene.

For the differentially expressed lncRNAs, we also used COXPH to study whether their expression levels were associated with the prognosis.

### Curated cancer-related lncRNAs

Curated liver cancer-related lncRNAs were collected through the database Lnc2Cancer, which is a manually curated database that provides comprehensive experimentally supported association between lncRNA and human cancer [5]. There were totally 10 liver cancer-related lncRNAs which were either up-regulated or down-regulated in patients, but only 2 lncRNAs appeared in our 9511 lncRNAs dataset. Some of them did not belong to the high confidence lncRNAs, the identification of some lncRNAs could be converted into ensemble gene id and others’ expression levels were unavailable. This suggested that this database curated manually some low confident lncRNAs. To compensate the few curated cancer-related lncRNAs, the lncRNAs listed in Su, Malouf [6] were also as a supplement.

### Functional analysis of co-expressed mRNAs

Since there were few studies about the functions of lncRNAs systematically, only a few lncRNAs had the functional annotations. To obtain the biological function of these lncRNAs, the co-expressed mRNAs in the liver cancer were figured out and the enriched GO terms and KEGG pathways were obtained. The correlation was calculated by linear regression and the cutoff for the coefficient and the P-value was set as 2 and 0.001, respectively [16]. The p-value of GO-term [17] and KEGG-pathway [18] enrichment was adjusted by Bonferroni corrections and 0.01 was used as the cutoff.

## SUPPLEMENTARY MATERIALS TABLES




