# Supplementary material for: DDX11-AS1 as potential therapy targets for human hepatocellular carcinoma
Source: Oncotarget. 2017 Apr 25;8(27):44195–202. doi: 10.18632/oncotarget.17409 (PMC5546473; doi:10.18632/oncotarget.17409)
Supplement: Supplementary file 1 [file oncotarget-08-44195-s001.pdf]

DDX11-AS1 as potential therapy targets for human hepatocellular carcinoma

SUPPLEMENTARY TABLES

Supplementary Table 1: Full tables of KEGG pathway enrichment

KEGG pathway			
**Accession**	**Pathway Name**	**P Value**	**Bonferroni**
hsa05200	Pathways In Cancer	0	0
hsa03040	Spliceosome	0	0
hsa05016	Huntingtons Disease	0	0
hsa04120	Ubiquitin Mediated Proteolysis	0	0
hsa03010	Ribosome	0	0
hsa00970	Aminoacyl Trna Biosynthesis	1.19E-12	2.15E-10
hsa04510	Focal Adhesion	4.51E-11	8.12E-09
hsa00240	Pyrimidine Metabolism	6.94E-11	1.24E-08
hsa04144	Endocytosis	1.02E-10	1.83E-08
hsa04110	Cell Cycle	1.08E-10	1.95E-08
hsa00230	Purine Metabolism	1.60E-10	2.89E-08
hsa04810	Regulation Of Actin Cytoskeleton	2.00E-10	3.61E-08
hsa03430	Mismatch Repair	3.63E-10	6.54E-08
hsa04722	Neurotrophin Signaling Pathway	4.76E-10	8.57E-08
hsa03030	Dna Replication	9.54E-10	1.71E-07
hsa03020	Rna Polymerase	1.60E-09	2.88E-07
hsa03018	Rna Degradation	6.25E-09	1.12E-06
hsa03420	Nucleotide Excision Repair	3.51E-08	6.33E-06
hsa04330	Notch Signaling Pathway	3.58E-08	6.44E-06
hsa03440	Homologous Recombination	5.71E-08	1.02E-05
hsa04114	Oocyte Meiosis	2.93E-07	5.29E-05
hsa05222	Small Cell Lung Cancer	7.48E-07	1.34E-04
hsa00310	Lysine Degradation	9.58E-07	1.72E-04
hsa03450	Non Homologous End Joining	1.09E-06	1.97E-04
hsa05120	Epithelial Cell Signaling In Helicobacter Pylori Infection	1.33E-06	2.41E-04
hsa04150	Mtor Signaling Pathway	1.96E-06	3.54E-04
hsa04910	Insulin Signaling Pathway	2.04E-06	3.68E-04
hsa05130	Pathogenic Escherichia Coli Infection	2.21E-06	3.98E-04
hsa05220	Chronic Myeloid Leukemia	2.26E-06	4.06E-04
hsa04512	Ecm Receptor Interaction	2.38E-06	4.29E-04
hsa05010	Alzheimers Disease	4.42E-06	7.95E-04
hsa05210	Colorectal Cancer	6.76E-06	0.001
hsa04520	Adherens Junction	7.91E-06	0.001
hsa04010	Mapk Signaling Pathway	8.15E-06	0.001
hsa05211	Renal Cell Carcinoma	9.03E-06	0.001
hsa04142	Lysosome	1.25E-05	0.002
hsa00563	Glycosylphosphatidylinositol Gpi Anchor Biosynthesis	1.74E-05	0.003
hsa04270	Vascular Smooth Muscle Contraction	1.97E-05	0.003
hsa04914	Progesterone Mediated Oocyte Maturation	3.58E-05	0.006
hsa04070	Phosphatidylinositol Signaling System	5.36E-05	0.009

Supplementary Table 2: Full tables of GO term enrichment

See Supplementary File 1

Supplementary Table 3: List of cancer-related lncRNAs presented in lncRNA pool

Symbol	Ensemble Gene ID	logFC	P Value	FDR	expression pattern	cancer type
PCA3	ENSG00000225937.1	1.18	1.2E-17	1.8E-15	up-regulated	prostate cancer
UCA1	ENSG00000214049.6	0.75	5.8E-08	1.2E-06	up-regualted	multiple cancers
KCNQ1OT1	ENSG00000269821.1	-0.59	2.0E-05	2.1E-04	differential expression	multiple cancers
MIR155HG	ENSG00000234883.3	-0.32	2.0E-02	6.8E-02	down-regulated	glioma
HULC	ENSG00000251164.1	-0.21	1.3E-01	2.7E-01	up-regulated	multiple cancers
PCAT1	ENSG00000253438.2	-0.14	3.0E-01	4.8E-01	up-regulated	prostate cancer
