# Supplementary material for: DDX11-AS1 as potential therapy targets for human hepatocellular carcinoma
Source: Oncotarget. 2017 Apr 25;8(27):44195–202. doi: 10.18632/oncotarget.17409 (PMC5546473; doi:10.18632/oncotarget.17409)
Supplement: Supplementary file 2 [file oncotarget-08-44195-s002.docx]

Accession Term Name P Value Bonferroni

#Biological Process

GO:0010467 gene expression 0 0

GO:0044267 cellular protein metabolic process 0 0

GO:0000278 mitotic cell cycle 0 0

GO:0045944 positive regulation of transcription from RNA polymerase II promoter 0 0

GO:0045087 innate immune response 0 0

GO:0000122 negative regulation of transcription from RNA polymerase II promoter 0 0

GO:0016032 viral process 0 0

GO:0045893 "positive regulation of transcription, DNA-templated" 0 0

GO:0019048 modulation by virus of host morphology or physiology 0 0

GO:0006281 DNA repair 0 0

GO:0015031 protein transport 0 0

GO:0016070 RNA metabolic process 0 0

GO:0008380 RNA splicing 0 0

GO:0006412 translation 0 0

GO:0007596 blood coagulation 0 0

GO:0016071 mRNA metabolic process 0 0

GO:0006366 transcription from RNA polymerase II promoter 0 0

GO:0000398 "mRNA splicing, via spliceosome" 0 0

GO:0006468 protein phosphorylation 0 0

GO:0007067 mitosis 0 0

GO:0016567 protein ubiquitination 0 0

GO:0048011 neurotrophin TRK receptor signaling pathway 0 0

GO:0006397 mRNA processing 0 0

GO:0006357 regulation of transcription from RNA polymerase II promoter 0 0

GO:0006367 transcription initiation from RNA polymerase II promoter 0 0

GO:0006413 translational initiation 0 0

GO:0000184 "nuclear-transcribed mRNA catabolic process, nonsense-mediated decay" 0 0

GO:0006260 DNA replication 0 0

GO:0001701 in utero embryonic development 0 0

GO:0006614 SRP-dependent cotranslational protein targeting to membrane 0 0

GO:0006414 translational elongation 0 0

GO:0019058 viral life cycle 0 0

GO:0006415 translational termination 0 0

GO:0006364 rRNA processing 0 0

GO:0019083 viral transcription 0 0

GO:0048205 COPI coating of Golgi vesicle 0 0

GO:0006189 'de novo' IMP biosynthetic process 0 0

GO:0031442 positive regulation of mRNA 3'-end processing 0 0

GO:0000462 "maturation of SSU-rRNA from tricistronic rRNA transcript (SSU-rRNA, 5.8S rRNA, LSU-rRNA)" 0 0

GO:0009113 purine nucleobase biosynthetic process 0 0

GO:0006734 NADH metabolic process 0 0

GO:0007176 regulation of epidermal growth factor-activated receptor activity 0 0

GO:0000389 mRNA 3'-splice site recognition 0 0

GO:0006482 protein demethylation 0 0

GO:0044387 negative regulation of protein kinase activity by regulation of protein phosphorylation 0 0

GO:0030174 regulation of DNA-dependent DNA replication initiation 0 0

GO:0046533 negative regulation of photoreceptor cell differentiation 0 0

GO:0006432 phenylalanyl-tRNA aminoacylation 0 0

GO:0030488 tRNA methylation 0 0

GO:0030490 maturation of SSU-rRNA 0 0

GO:0000447 "endonucleolytic cleavage in ITS1 to separate SSU-rRNA from 5.8S rRNA and LSU-rRNA from tricistronic rRNA transcript (SSU-rRNA, 5.8S rRNA, LSU-rRNA)" 0 0

GO:0007221 positive regulation of transcription of Notch receptor target 0 0

GO:0043631 RNA polyadenylation 0 0

GO:0007161 calcium-independent cell-matrix adhesion 0 0

GO:0009912 auditory receptor cell fate commitment 0 0

GO:0035246 peptidyl-arginine N-methylation 0 0

GO:0060178 regulation of exocyst localization 0 0

GO:0006434 seryl-tRNA aminoacylation 0 0

GO:0048340 paraxial mesoderm morphogenesis 0 0

GO:0061014 positive regulation of mRNA catabolic process 0 0

GO:0036066 protein O-linked fucosylation 0 0

GO:0036265 RNA (guanine-N7)-methylation 0 0

GO:0061084 negative regulation of protein refolding 0 0

GO:0048597 post-embryonic camera-type eye morphogenesis 0 0

GO:0061198 fungiform papilla formation 0 0

GO:0010248 establishment or maintenance of transmembrane electrochemical gradient 0 0

GO:0007079 mitotic chromosome movement towards spindle pole 0 0

GO:0050856 regulation of T cell receptor signaling pathway 0 0

GO:0042524 negative regulation of tyrosine phosphorylation of Stat5 protein 0 0

GO:0010424 DNA methylation on cytosine within a CG sequence 0 0

GO:0051088 PMA-inducible membrane protein ectodomain proteolysis 0 0

GO:0072144 glomerular mesangial cell development 0 0

GO:0072197 ureter morphogenesis 0 0

GO:0010457 centriole-centriole cohesion 0 0

GO:0072673 lamellipodium morphogenesis 0 0

GO:0034201 response to oleic acid 0 0

GO:0097039 protein linear polyubiquitination 0 0

GO:0097062 dendritic spine maintenance 0 0

GO:1990126 "retrograde transport, endosome to plasma membrane" 0 0

GO:0051683 establishment of Golgi localization 0 0

GO:0003256 regulation of transcription from RNA polymerase II promoter involved in myocardial precursor cell differentiation 0 0

GO:0010834 telomere maintenance via telomere shortening 0 0

GO:0051877 pigment granule aggregation in cell center 0 0

GO:0006283 transcription-coupled nucleotide-excision repair 2.22E-16 1.29E-15

GO:0006396 RNA processing 5.55E-16 3.23E-15

GO:0050434 positive regulation of viral transcription 5.55E-16 3.23E-15

GO:0006464 cellular protein modification process 1.03E-14 6.00E-14

GO:0007077 mitotic nuclear envelope disassembly 1.77E-14 1.03E-13

GO:0006418 tRNA aminoacylation for protein translation 2.77E-14 1.61E-13

GO:0006289 nucleotide-excision repair 6.73E-14 3.90E-13

GO:0006378 mRNA polyadenylation 1.70E-13 9.88E-13

GO:0006368 transcription elongation from RNA polymerase II promoter 3.82E-13 2.21E-12

GO:0051726 regulation of cell cycle 4.26E-13 2.46E-12

GO:0051028 mRNA transport 4.69E-13 2.70E-12

GO:0006370 7-methylguanosine mRNA capping 5.74E-13 3.31E-12

GO:0006338 chromatin remodeling 7.33E-13 4.22E-12

GO:0006626 protein targeting to mitochondrion 1.66E-12 9.59E-12

GO:0000387 spliceosomal snRNP assembly 3.84E-12 2.20E-11

GO:0032481 positive regulation of type I interferon production 7.07E-12 4.06E-11

GO:0006446 regulation of translational initiation 7.21E-12 4.14E-11

GO:0006200 ATP catabolic process 1.45E-11 8.31E-11

GO:0006915 apoptotic process 1.68E-11 9.64E-11

GO:0007179 transforming growth factor beta receptor signaling pathway 2.11E-11 1.21E-10

GO:0006302 double-strand break repair 2.28E-11 1.30E-10

GO:0042254 ribosome biogenesis 2.41E-11 1.37E-10

GO:0006987 activation of signaling protein activity involved in unfolded protein response 2.42E-11 1.38E-10

GO:0007049 cell cycle 2.45E-11 1.39E-10

GO:0045892 "negative regulation of transcription, DNA-templated" 2.55E-11 1.45E-10

GO:0044281 small molecule metabolic process 2.86E-11 1.62E-10

GO:0006351 "transcription, DNA-templated" 3.05E-11 1.73E-10

GO:0000209 protein polyubiquitination 3.34E-11 1.89E-10

GO:0006355 "regulation of transcription, DNA-templated" 3.46E-11 1.96E-10

GO:0006406 mRNA export from nucleus 4.20E-11 2.37E-10

GO:0006886 intracellular protein transport 4.57E-11 2.58E-10

GO:0006457 protein folding 4.60E-11 2.59E-10

GO:0006974 cellular response to DNA damage stimulus 5.00E-11 2.82E-10

GO:0000086 G2/M transition of mitotic cell cycle 6.35E-11 3.58E-10

GO:0007094 mitotic spindle assembly checkpoint 6.63E-11 3.73E-10

GO:0016192 vesicle-mediated transport 7.70E-11 4.33E-10

GO:0000289 nuclear-transcribed mRNA poly(A) tail shortening 8.05E-11 4.52E-10

GO:0034660 ncRNA metabolic process 9.47E-11 5.31E-10

GO:0010827 regulation of glucose transport 9.90E-11 5.55E-10

GO:0043065 positive regulation of apoptotic process 1.09E-10 6.11E-10

GO:0006184 GTP catabolic process 1.21E-10 6.77E-10

GO:0000082 G1/S transition of mitotic cell cycle 1.42E-10 7.95E-10

GO:0042059 negative regulation of epidermal growth factor receptor signaling pathway 1.70E-10 9.53E-10

GO:0030968 endoplasmic reticulum unfolded protein response 1.79E-10 1.00E-09

GO:0007173 epidermal growth factor receptor signaling pathway 1.95E-10 1.09E-09

GO:0031124 mRNA 3'-end processing 2.12E-10 1.18E-09

GO:0007264 small GTPase mediated signal transduction 2.21E-10 1.23E-09

GO:0006417 regulation of translation 2.33E-10 1.29E-09

GO:0000724 double-strand break repair via homologous recombination 3.50E-10 1.94E-09

GO:0008150 biological_process 4.01E-10 2.22E-09

GO:0007059 chromosome segregation 4.08E-10 2.26E-09

GO:0006363 termination of RNA polymerase I transcription 4.41E-10 2.44E-09

GO:0006369 termination of RNA polymerase II transcription 5.31E-10 2.93E-09

GO:0051301 cell division 6.31E-10 3.48E-09

GO:0030198 extracellular matrix organization 6.96E-10 3.84E-09

GO:0034138 toll-like receptor 3 signaling pathway 7.93E-10 4.36E-09

GO:0031145 anaphase-promoting complex-dependent proteasomal ubiquitin-dependent protein catabolic process 7.93E-10 4.36E-09

GO:0008285 negative regulation of cell proliferation 8.47E-10 4.66E-09

GO:0006360 transcription from RNA polymerase I promoter 8.65E-10 4.75E-09

GO:0006310 DNA recombination 1.04E-09 5.74E-09

GO:0006361 transcription initiation from RNA polymerase I promoter 1.21E-09 6.64E-09

GO:0051439 regulation of ubiquitin-protein ligase activity involved in mitotic cell cycle 1.52E-09 8.35E-09

GO:0007411 axon guidance 1.61E-09 8.81E-09

GO:0007220 Notch receptor processing 1.62E-09 8.85E-09

GO:0043967 histone H4 acetylation 1.62E-09 8.85E-09

GO:0034162 toll-like receptor 9 signaling pathway 1.66E-09 9.08E-09

GO:0001525 angiogenesis 1.76E-09 9.62E-09

GO:0002224 toll-like receptor signaling pathway 1.83E-09 9.97E-09

GO:0006511 ubiquitin-dependent protein catabolic process 2.14E-09 1.16E-08

GO:0035666 TRIF-dependent toll-like receptor signaling pathway 2.40E-09 1.30E-08

GO:0043161 proteasome-mediated ubiquitin-dependent protein catabolic process 2.94E-09 1.59E-08

GO:0007507 heart development 4.12E-09 2.23E-08

GO:0051084 'de novo' posttranslational protein folding 4.42E-09 2.39E-08

GO:0051437 positive regulation of ubiquitin-protein ligase activity involved in mitotic cell cycle 4.69E-09 2.54E-08

GO:0010212 response to ionizing radiation 5.25E-09 2.83E-08

GO:0034166 toll-like receptor 10 signaling pathway 5.58E-09 3.01E-08

GO:0051436 negative regulation of ubiquitin-protein ligase activity involved in mitotic cell cycle 5.58E-09 3.01E-08

GO:0034146 toll-like receptor 5 signaling pathway 5.58E-09 3.01E-08

GO:0002756 MyD88-independent toll-like receptor signaling pathway 5.80E-09 3.12E-08

GO:0032508 DNA duplex unwinding 7.13E-09 3.84E-08

GO:0008283 cell proliferation 8.81E-09 4.73E-08

GO:0034142 toll-like receptor 4 signaling pathway 8.98E-09 4.82E-08

GO:0007165 signal transduction 9.01E-09 4.83E-08

GO:0006362 transcription elongation from RNA polymerase I promoter 9.28E-09 4.97E-08

GO:0006271 DNA strand elongation involved in DNA replication 9.41E-09 5.03E-08

GO:0042274 ribosomal small subunit biogenesis 9.81E-09 5.24E-08

GO:0030036 actin cytoskeleton organization 1.03E-08 5.53E-08

GO:0016568 chromatin modification 1.08E-08 5.78E-08

GO:0001731 formation of translation preinitiation complex 1.16E-08 6.20E-08

GO:0051568 histone H3-K4 methylation 1.16E-08 6.20E-08

GO:0000723 telomere maintenance 1.16E-08 6.20E-08

GO:0042981 regulation of apoptotic process 1.16E-08 6.20E-08

GO:0034134 toll-like receptor 2 signaling pathway 1.16E-08 6.19E-08

GO:0006890 "retrograde vesicle-mediated transport, Golgi to ER" 1.48E-08 7.86E-08

GO:0007219 Notch signaling pathway 1.58E-08 8.37E-08

GO:0006383 transcription from RNA polymerase III promoter 1.75E-08 9.31E-08

GO:0009411 response to UV 1.92E-08 1.01E-07

GO:0038124 toll-like receptor TLR6:TLR2 signaling pathway 2.01E-08 1.06E-07

GO:0038123 toll-like receptor TLR1:TLR2 signaling pathway 2.01E-08 1.06E-07

GO:0000288 "nuclear-transcribed mRNA catabolic process, deadenylation-dependent decay" 2.02E-08 1.06E-07

GO:0002755 MyD88-dependent toll-like receptor signaling pathway 2.98E-08 1.57E-07

GO:0071230 cellular response to amino acid stimulus 3.33E-08 1.75E-07

GO:0008033 tRNA processing 3.54E-08 1.86E-07

GO:0030100 regulation of endocytosis 4.17E-08 2.19E-07

GO:0007050 cell cycle arrest 4.65E-08 2.44E-07

GO:0006303 double-strand break repair via nonhomologous end joining 5.20E-08 2.73E-07

GO:0051403 stress-activated MAPK cascade 5.70E-08 2.98E-07

GO:0009168 purine ribonucleoside monophosphate biosynthetic process 5.75E-08 3.01E-07

GO:0043123 positive regulation of I-kappaB kinase/NF-kappaB signaling 5.84E-08 3.05E-07

GO:0008645 hexose transport 6.14E-08 3.20E-07

GO:0031175 neuron projection development 7.02E-08 3.66E-07

GO:0008286 insulin receptor signaling pathway 7.57E-08 3.95E-07

GO:0000910 cytokinesis 7.71E-08 4.01E-07

GO:0016477 cell migration 8.77E-08 4.56E-07

GO:0051297 centrosome organization 9.07E-08 4.71E-07

GO:0070979 protein K11-linked ubiquitination 9.32E-08 4.84E-07

GO:0007266 Rho protein signal transduction 1.09E-07 5.66E-07

GO:0006306 DNA methylation 1.32E-07 6.86E-07

GO:0000245 spliceosomal complex assembly 1.32E-07 6.86E-07

GO:0043984 histone H4-K16 acetylation 1.32E-07 6.86E-07

GO:0016197 endosomal transport 1.35E-07 6.98E-07

GO:0030168 platelet activation 1.61E-07 8.32E-07

GO:0006977 "DNA damage response, signal transduction by p53 class mediator resulting in cell cycle arrest" 1.62E-07 8.38E-07

GO:0046777 protein autophosphorylation 1.68E-07 8.67E-07

GO:0043966 histone H3 acetylation 1.92E-07 9.92E-07

GO:0043982 histone H4-K8 acetylation 2.41E-07 1.23E-06

GO:0043981 histone H4-K5 acetylation 2.41E-07 1.23E-06

GO:0009790 embryo development 2.49E-07 1.28E-06

GO:0007265 Ras protein signal transduction 2.63E-07 1.35E-06

GO:0019985 translesion synthesis 2.68E-07 1.37E-06

GO:0018279 protein N-linked glycosylation via asparagine 3.00E-07 1.53E-06

GO:0006606 protein import into nucleus 3.01E-07 1.54E-06

GO:0033572 transferrin transport 5.21E-07 2.66E-06

GO:0001837 epithelial to mesenchymal transition 5.21E-07 2.66E-06

GO:0005975 carbohydrate metabolic process 5.22E-07 2.66E-06

GO:0051260 protein homooligomerization 5.27E-07 2.69E-06

GO:0043066 negative regulation of apoptotic process 5.48E-07 2.79E-06

GO:0006385 transcription elongation from RNA polymerase III promoter 5.58E-07 2.83E-06

GO:0006386 termination of RNA polymerase III transcription 5.58E-07 2.83E-06

GO:0000722 telomere maintenance via recombination 7.57E-07 3.84E-06

GO:0051056 regulation of small GTPase mediated signal transduction 7.84E-07 3.97E-06

GO:0042384 cilium assembly 9.08E-07 4.60E-06

GO:0032469 endoplasmic reticulum calcium ion homeostasis 9.35E-07 4.72E-06

GO:0033523 histone H2B ubiquitination 9.35E-07 4.72E-06

GO:0042769 "DNA damage response, detection of DNA damage" 9.35E-07 4.72E-06

GO:0051292 nuclear pore complex assembly 9.35E-07 4.72E-06

GO:0035556 intracellular signal transduction 9.46E-07 4.77E-06

GO:0030512 negative regulation of transforming growth factor beta receptor signaling pathway 1.08E-06 5.48E-06

GO:0055086 nucleobase-containing small molecule metabolic process 1.09E-06 5.53E-06

GO:0015758 glucose transport 1.27E-06 6.41E-06

GO:0006611 protein export from nucleus 1.27E-06 6.41E-06

GO:0043968 histone H2A acetylation 1.35E-06 6.79E-06

GO:0043687 post-translational protein modification 1.40E-06 7.05E-06

GO:0051291 protein heterooligomerization 1.42E-06 7.11E-06

GO:0008219 cell death 1.54E-06 7.72E-06

GO:0007030 Golgi organization 1.68E-06 8.45E-06

GO:0045732 positive regulation of protein catabolic process 1.78E-06 8.92E-06

GO:0032436 positive regulation of proteasomal ubiquitin-dependent protein catabolic process 1.83E-06 9.18E-06

#Molecular Function

GO:0005515 protein binding 0 0

GO:0003677 DNA binding 0 0

GO:0005524 ATP binding 0 0

GO:0003723 RNA binding 0 0

GO:0003700 sequence-specific DNA binding transcription factor activity 0 0

GO:0003674 molecular_function 0 0

GO:0003682 chromatin binding 0 0

GO:0042802 identical protein binding 0 0

GO:0005525 GTP binding 0 0

GO:0019901 protein kinase binding 0 0

GO:0004842 ubiquitin-protein ligase activity 0 0

GO:0008134 transcription factor binding 0 0

GO:0019899 enzyme binding 0 0

GO:0003735 structural constituent of ribosome 0 0

GO:0004672 protein kinase activity 0 0

GO:0003714 transcription corepressor activity 0 0

GO:0019904 protein domain specific binding 0 0

GO:0003923 GPI-anchor transamidase activity 0 0

GO:0070300 phosphatidic acid binding 0 0

GO:0019887 protein kinase regulator activity 0 0

GO:0032137 guanine/thymine mispair binding 0 0

GO:0032549 ribonucleoside binding 0 0

GO:0032564 dATP binding 0 0

GO:0016274 protein-arginine N-methyltransferase activity 0 0

GO:0045322 unmethylated CpG binding 0 0

GO:0046976 histone methyltransferase activity (H3-K27 specific) 0 0

GO:0032139 dinucleotide insertion or deletion binding 0 0

GO:0032142 single guanine insertion binding 0 0

GO:0032357 oxidized purine DNA binding 0 0

GO:0004828 serine-tRNA ligase activity 0 0

GO:0010485 H4 histone acetyltransferase activity 0 0

GO:0043139 5'-3' DNA helicase activity 0 0

GO:0004706 JUN kinase kinase kinase activity 0 0

GO:0017160 Ral GTPase binding 0 0

GO:0034648 histone demethylase activity (H3-dimethyl-K4 specific) 0 0

GO:0002134 UTP binding 0 0

GO:0030942 endoplasmic reticulum signal peptide binding 0 0

GO:0071535 RING-like zinc finger domain binding 0 0

GO:0097157 pre-mRNA intronic binding 0 0

GO:1990226 histone methyltransferase binding 0 0

GO:0003743 translation initiation factor activity 1.37E-14 5.72E-14

GO:0000166 nucleotide binding 6.71E-12 2.78E-11

GO:0008270 zinc ion binding 1.19E-11 4.93E-11

GO:0004674 protein serine/threonine kinase activity 2.19E-11 9.07E-11

GO:0046872 metal ion binding 2.32E-11 9.60E-11

GO:0003899 DNA-directed RNA polymerase activity 3.45E-11 1.42E-10

GO:0003713 transcription coactivator activity 4.80E-11 1.96E-10

GO:0042803 protein homodimerization activity 4.80E-11 1.96E-10

GO:0042393 histone binding 7.57E-11 3.09E-10

GO:0003924 GTPase activity 7.62E-11 3.11E-10

GO:0003729 mRNA binding 7.65E-11 3.11E-10

GO:0031625 ubiquitin protein ligase binding 8.71E-11 3.53E-10

GO:0003676 nucleic acid binding 1.15E-10 4.68E-10

GO:0016887 ATPase activity 1.21E-10 4.89E-10

GO:0008022 protein C-terminus binding 1.60E-10 6.49E-10

GO:0008026 ATP-dependent helicase activity 4.08E-10 1.64E-09

GO:0004003 ATP-dependent DNA helicase activity 5.97E-10 2.39E-09

GO:0008094 DNA-dependent ATPase activity 6.59E-10 2.64E-09

GO:0017124 SH3 domain binding 7.97E-10 3.18E-09

GO:0051082 unfolded protein binding 8.59E-10 3.43E-09

GO:0000049 tRNA binding 9.54E-10 3.80E-09

GO:0003697 single-stranded DNA binding 1.13E-09 4.52E-09

GO:0031593 polyubiquitin binding 1.62E-09 6.42E-09

GO:0003678 DNA helicase activity 2.03E-09 8.06E-09

GO:0047485 protein N-terminus binding 2.55E-09 1.00E-08

GO:0008013 beta-catenin binding 3.57E-09 1.40E-08

GO:0003725 double-stranded RNA binding 3.42E-08 1.34E-07

GO:0003684 damaged DNA binding 6.21E-08 2.44E-07

GO:0004402 histone acetyltransferase activity 6.27E-08 2.45E-07

GO:0000287 magnesium ion binding 7.08E-08 2.77E-07

GO:0008536 Ran GTPase binding 9.32E-08 3.63E-07

GO:0008565 protein transporter activity 1.20E-07 4.69E-07

GO:0051015 actin filament binding 1.29E-07 5.03E-07

GO:0046972 histone acetyltransferase activity (H4-K16 specific) 1.64E-07 6.36E-07

GO:0043995 histone acetyltransferase activity (H4-K5 specific) 1.64E-07 6.36E-07

GO:0043996 histone acetyltransferase activity (H4-K8 specific) 1.64E-07 6.36E-07

GO:0005487 nucleocytoplasmic transporter activity 2.80E-07 1.08E-06

GO:0001104 RNA polymerase II transcription cofactor activity 3.01E-07 1.15E-06

GO:0030331 estrogen receptor binding 7.57E-07 2.90E-06

GO:0004004 ATP-dependent RNA helicase activity 9.28E-07 3.55E-06

GO:0042054 histone methyltransferase activity 9.35E-07 3.56E-06

GO:0034450 ubiquitin-ubiquitin ligase activity 9.35E-07 3.56E-06

GO:0030374 ligand-dependent nuclear receptor transcription coactivator activity 1.01E-06 3.85E-06

GO:0030234 enzyme regulator activity 1.09E-06 4.17E-06

GO:0008168 methyltransferase activity 1.12E-06 4.25E-06

GO:0032403 protein complex binding 1.18E-06 4.49E-06

GO:0008135 "translation factor activity, nucleic acid binding" 1.27E-06 4.81E-06

GO:0042800 histone methyltransferase activity (H3-K4 specific) 1.35E-06 5.09E-06

GO:0043130 ubiquitin binding 2.98E-06 1.12E-05

GO:0032947 protein complex scaffold 3.02E-06 1.13E-05

GO:0046982 protein heterodimerization activity 3.30E-06 1.23E-05

GO:0051087 chaperone binding 4.39E-06 1.64E-05

GO:0051539 "4 iron, 4 sulfur cluster binding" 5.26E-06 1.96E-05

GO:0019237 centromeric DNA binding 5.30E-06 1.97E-05

GO:0044212 transcription regulatory region DNA binding 5.53E-06 2.05E-05

GO:0015485 cholesterol binding 5.72E-06 2.12E-05

#Cellular Component

GO:0005634 nucleus 0 0

GO:0005829 cytosol 0 0

GO:0005730 nucleolus 0 0

GO:0005654 nucleoplasm 0 0

GO:0005739 mitochondrion 0 0

GO:0016020 membrane 0 0

GO:0048471 perinuclear region of cytoplasm 0 0

GO:0043231 intracellular membrane-bounded organelle 0 0

GO:0016607 nuclear speck 0 0

GO:0043234 protein complex 0 0

GO:0030529 ribonucleoprotein complex 0 0

GO:0031965 nuclear membrane 0 0

GO:0005819 spindle 0 0

GO:0005681 spliceosomal complex 0 0

GO:0071013 catalytic step 2 spliceosome 0 0

GO:0016363 nuclear matrix 0 0

GO:0072357 PTW/PP1 phosphatase complex 0 0

GO:0042719 mitochondrial intermembrane space protein transporter complex 0 0

GO:0042765 GPI-anchor transamidase complex 0 0

GO:0000109 nucleotide-excision repair complex 0 0

GO:0030686 90S preribosome 0 0

GO:0044615 nuclear pore nuclear basket 0 0

GO:0031428 box C/D snoRNP complex 0 0

GO:0005862 muscle thin filament tropomyosin 0 0

GO:0097452 GAIT complex 0 0

GO:0044530 supraspliceosomal complex 0 0

GO:0000938 GARP complex 0 0

GO:0031618 nuclear centromeric heterochromatin 0 0

GO:0009331 glycerol-3-phosphate dehydrogenase complex 0 0

GO:0000942 condensed nuclear chromosome outer kinetochore 0 0

GO:0002193 MAML1-RBP-Jkappa- ICN1 complex 0 0

GO:0032449 CBM complex 0 0

GO:0071437 invadopodium 0 0

GO:0005850 eukaryotic translation initiation factor 2 complex 0 0

GO:0071797 LUBAC complex 0 0

GO:0043259 laminin-10 complex 0 0

GO:0016939 kinesin II complex 0 0

GO:0097149 centralspindlin complex 0 0

GO:0022625 cytosolic large ribosomal subunit 3.33E-16 2.09E-15

GO:0005694 chromosome 6.66E-16 4.15E-15

GO:0030496 midbody 3.44E-15 2.12E-14

GO:0071339 MLL1 complex 7.77E-15 4.77E-14

GO:0000776 kinetochore 8.04E-14 4.91E-13

GO:0005737 cytoplasm 4.71E-13 2.85E-12

GO:0000775 "chromosome, centromeric region" 2.19E-12 1.32E-11

GO:0022627 cytosolic small ribosomal subunit 1.09E-11 6.57E-11

GO:0005794 Golgi apparatus 1.53E-11 9.11E-11

GO:0000139 Golgi membrane 2.22E-11 1.31E-10

GO:0005789 endoplasmic reticulum membrane 2.25E-11 1.32E-10

GO:0015629 actin cytoskeleton 2.27E-11 1.32E-10

GO:0005813 centrosome 3.11E-11 1.80E-10

GO:0005643 nuclear pore 4.20E-11 2.41E-10

GO:0015030 Cajal body 4.27E-11 2.44E-10

GO:0005783 endoplasmic reticulum 4.53E-11 2.57E-10

GO:0005765 lysosomal membrane 6.58E-11 3.71E-10

GO:0005769 early endosome 9.90E-11 5.54E-10

GO:0035097 histone methyltransferase complex 4.41E-10 2.45E-09

GO:0042470 melanosome 4.84E-10 2.68E-09

GO:0005856 cytoskeleton 5.15E-10 2.83E-09

GO:0000145 exocyst 1.85E-09 1.01E-08

GO:0005874 microtubule 1.92E-09 1.04E-08

GO:0000922 spindle pole 3.92E-09 2.11E-08

GO:0005840 ribosome 5.11E-09 2.74E-08

GO:0005743 mitochondrial inner membrane 5.28E-09 2.81E-08

GO:0070062 extracellular vesicular exosome 6.58E-09 3.48E-08

GO:0030027 lamellipodium 7.74E-09 4.08E-08

GO:0000777 condensed chromosome kinetochore 8.24E-09 4.31E-08

GO:0000123 histone acetyltransferase complex 1.11E-08 5.77E-08

GO:0005665 "DNA-directed RNA polymerase II, core complex" 1.16E-08 6.00E-08

GO:0016282 eukaryotic 43S preinitiation complex 1.16E-08 6.00E-08

GO:0005689 U12-type spliceosomal complex 1.48E-08 7.58E-08

GO:0000785 chromatin 1.57E-08 8.01E-08

GO:0017053 transcriptional repressor complex 2.04E-08 1.03E-07

GO:0016592 mediator complex 2.60E-08 1.31E-07

GO:0032040 small-subunit processome 2.90E-08 1.45E-07

GO:0005925 focal adhesion 3.03E-08 1.51E-07

GO:0005815 microtubule organizing center 3.13E-08 1.55E-07

GO:0005635 nuclear envelope 3.24E-08 1.59E-07

GO:0005852 eukaryotic translation initiation factor 3 complex 5.20E-08 2.54E-07

GO:0035267 NuA4 histone acetyltransferase complex 5.75E-08 2.78E-07

GO:0033290 eukaryotic 48S preinitiation complex 5.75E-08 2.78E-07

GO:0031902 late endosome membrane 7.54E-08 3.62E-07

GO:0005604 basement membrane 1.21E-07 5.81E-07

GO:0005832 chaperonin-containing T-complex 1.64E-07 7.76E-07

GO:0042405 nuclear inclusion body 1.64E-07 7.76E-07

GO:0005847 mRNA cleavage and polyadenylation specificity factor complex 1.64E-07 7.76E-07

GO:0005788 endoplasmic reticulum lumen 1.73E-07 8.12E-07

GO:0016581 NuRD complex 1.85E-07 8.62E-07

GO:0005667 transcription factor complex 1.88E-07 8.74E-07

GO:0030014 CCR4-NOT complex 2.41E-07 1.11E-06

GO:0031012 extracellular matrix 3.33E-07 1.53E-06

GO:0000794 condensed nuclear chromosome 3.48E-07 1.59E-06

GO:0000151 ubiquitin ligase complex 3.68E-07 1.67E-06

GO:0015630 microtubule cytoskeleton 4.36E-07 1.97E-06

GO:0030532 small nuclear ribonucleoprotein complex 5.58E-07 2.50E-06

GO:0010008 endosome membrane 6.68E-07 2.98E-06

GO:0005732 small nucleolar ribonucleoprotein complex 9.35E-07 4.13E-06

GO:0043596 nuclear replication fork 9.35E-07 4.13E-06

GO:0008180 COP9 signalosome 1.04E-06 4.58E-06

GO:0032587 ruffle membrane 1.08E-06 4.76E-06

GO:0005736 DNA-directed RNA polymerase I complex 1.38E-06 5.97E-06

GO:0035861 site of double-strand break 1.38E-06 5.97E-06

GO:0031080 nuclear pore outer ring 1.38E-06 5.97E-06

GO:0005575 cellular_component 1.39E-06 5.97E-06

GO:0000502 proteasome complex 1.71E-06 7.34E-06

GO:0042645 mitochondrial nucleoid 1.78E-06 7.60E-06

GO:0000781 "chromosome, telomeric region" 1.83E-06 7.78E-06

GO:0005911 cell-cell junction 2.11E-06 8.91E-06

GO:0005666 DNA-directed RNA polymerase III complex 3.45E-06 1.44E-05

GO:0051233 spindle midzone 3.45E-06 1.44E-05

GO:0005759 mitochondrial matrix 4.09E-06 1.69E-05

GO:0000346 transcription export complex 4.92E-06 2.03E-05

GO:0031252 cell leading edge 5.27E-06 2.16E-05

GO:0016593 Cdc73/Paf1 complex 5.30E-06 2.16E-05

GO:0030130 clathrin coat of trans-Golgi network vesicle 5.30E-06 2.16E-05

GO:0002102 podosome 5.79E-06 2.34E-05

GO:0005720 nuclear heterochromatin 5.79E-06 2.34E-05

GO:0005675 holo TFIIH complex 6.42E-06 2.57E-05

GO:0002199 zona pellucida receptor complex 6.42E-06 2.57E-05

GO:0000940 condensed chromosome outer kinetochore 7.09E-06 2.80E-05

GO:0046930 pore complex 7.09E-06 2.80E-05

GO:0048188 Set1C/COMPASS complex 7.09E-06 2.80E-05

GO:0005680 anaphase-promoting complex 7.78E-06 3.06E-05

GO:0005671 Ada2/Gcn5/Ada3 transcription activator complex 9.29E-06 3.63E-05

GO:0005876 spindle microtubule 9.86E-06 3.84E-05

GO:0010494 cytoplasmic stress granule 1.01E-05 3.92E-05

GO:0030426 growth cone 1.20E-05 4.64E-05
